# A Conversation with Craig McDonald, MD, Chair, Department of Physical Medicine & Rehabilitation, Regents of the University of California (Davis)

**DOI:** 10.1017/cts.2023.664

**Published:** 2023-12-11

**Authors:** Clinical Research Forum

**Affiliations:** Clinical Research Forum, Washington, DC, USA

## Top 10 Clinical Research Achievement Awards Q & A

This article is part of a series of interviews with recipients of Clinical Research Forum’s Top 10 Clinical Research Achievement Awards. This article is with Dr Craig McDonald, MD, Chair, Department of Physical Medicine & Rehabilitation, Regents of the University of California (Davis). Dr McDonald is a pediatric physical medicine and rehabilitation physician, and his research focuses on the development of novel therapeutics for neuromuscular diseases. Dr McDonald received a 2023 Top 10 Clinical Research Achievement Award for *Repeated intravenous cardiosphere-derived cell therapy in late-stage Duchenne muscular dystrophy (HOPE-2): a multicentre, randomised, double-blind, placebo-controlled, phase 2 trial. The interview has been edited for length and clarity.*

## When did you first become interested in pursuing a career in medicine?

My interest in medicine stems from when I was an undergraduate at Stanford and volunteered in a program to help patients with physical and developmental disabilities. That experience was incredibly rewarding, and it led me toward the field of rehabilitation medicine. Then, while I was a medical student, I found I also enjoyed learning about the nervous and musculoskeletal systems, particularly with regard to development, which helped me gravitate to pediatrics.

## How has the field of pediatric physical medicine and rehabilitation changed during your career?

When I started out, the focus was on the medical management of neuromuscular diseases and disorders, using primarily supportive care and trying to prevent medical complications. But over the past three decades, there’s been a remarkable transformation. We’ve seen an evolution where, with modern precision medicine therapeutics, we can actually target the underlying genetic causes of some of the inherited musculoskeletal conditions that affect both children and adults. The advances have been staggering. For instance, when I was a medical student I never dreamed we’d be treating patients with Duchenne muscular dystrophy with gene therapies and stem cell therapies. The gene for dystrophin protein which, if altered, is the underlying cause of this most common form of muscular dystrophy was first identified in 1986. Then, researchers identified the protein, which led to improved diagnostics. But now, we’re developing increasingly advanced therapeutic strategies – and they’re having a phenomenal impact on both survival and functional outcomes for patients. It has been extremely gratifying to be a part of this transformation in medicine.

## What does your award-winning trial show?

We found that cardiosphere-derived cells (CDCs) infused intravenously appear to be a safe and effective therapy for reducing deterioration of upper limb function in patients with late-stage Duchenne muscular dystrophy. This was a tremendous finding because these are patients who have developed progressive quadriplegia, and historically, they have not been included in trials of muscular dystrophy therapeutics because the thinking was that they were too impaired to experience benefits. These patients also have progressive cardiomyopathy, and in addition to reductions in the deterioration of upper limb function, our trial found that certain measures of cardiac function and structure were improved in the study group compared with the placebo group.

## What is the cellular mechanism involved?

The IV-infused CDCs secrete membrane-bound vesicles called exosomes, and these exosomes contain immunomodulatory components that reduce inflammation and improve regeneration of muscle tissue. The patient’s muscle tissue benefits from the systemic exposure to these molecules and shows reduced degeneration, inflammation, scar replacement, and regeneration. When our research with CDCs began, we originally targeted heart muscle with infusions via coronary artery catheterizations. What we discovered is not only did these infusions have a positive benefit on reducing scar formation in the heart, but the patients also experienced improvements off target, in their arm function. So, of course we wanted to see if infusing the cells intravenously could result in similar benefits – and that’s exactly what we found. This trial showed benefits in terms of the preservation of arm function as well as stabilization in cardiac deterioration.

## Where does this research go from here and how do cell-based therapies like this one compare to gene therapies?

Each therapeutic strategy has a role in the treatment of this condition. Generally speaking, gene therapy targets younger patients, typically 4 to 5 years old. It’s a one-time infusion and if done early, before much muscle tissue is lost, can help stabilize the disease or even provide a level of peak attained function which then continues to stabilize over time. Right now, we don’t know if gene therapy will have similar benefits in older patients. But in older patients, we’ve been able to use cell-based therapies and actually enhance and stabilize the deterioration that these patients experience in muscles of both their arms and their hearts. Ultimately, we may see a combination of gene therapy and cell-based approaches: gene therapy to treat the underlying cause of the disease by replacing the defective gene and getting the dystrophin protein into the muscle and then cell-based approaches to further enhance the function of the muscle and decrease the inflammation and immune-mediated deterioration of muscle function. This is similar to approaches for treating patients with cancer, many of whom get different forms of chemotherapy to attack different aspects of the cancer.

## The field of pediatric physical medicine and rehabilitation has evolved dramatically. Has your approach to clinical research changed over the years as well?

Throughout most of my career, I worked on clinical research aspects of several different childhood disabling conditions and it was gratifying to make contributions to our understanding of disorders such as spina bifida, cerebral palsy, traumatic brain injury, and so forth. But as I advanced in my career and took on more leadership responsibilities in the UC Davis Health System, I found I couldn’t continue with such a broad approach. Instead, I had to focus. That’s what’s enabled me to make more meaningful contributions to muscular dystrophy, including describing the natural history of the disease, developing clinical outcome measures to be used in clinical trials, and conducting studies like this one.

## What do you like most about your job?

Working in the field of rehabilitation medicine allows me to focus on issues related to improving the quality of life and maximizing functional performance for my patients and that’s really compelling to me. Also, as someone who specializes in pediatric physical medicine, I’m able to develop relationships with my patients from a very young age, often from the time they are infants or young children up until the time they graduate from high school and transition to adult activities. I get to know them and their families and it’s so rewarding to help a patient with physical or cognitive challenges over the years and then get photos of, or even invitations to, events like their high school graduation. In addition, I’m actively engaged in the patient advocacy and clinical translational research communities, not just here in the USA but internationally as well. That’s enabled me to develop professional collaborations – and friendships – with researchers all over the globe, and it’s been exciting to work with these colleagues and see the impact new therapies are having on patients.

